# A method for estimating yield of maize inbred lines by assimilating WOFOST model with Sentinel-2 satellite data

**DOI:** 10.3389/fpls.2023.1201179

**Published:** 2023-09-07

**Authors:** Junyi Liu, Xianpeng Hou, Shuaiming Chen, Yanhua Mu, Hai Huang, Hengbin Wang, Zhe Liu, Shaoming Li, Xiaodong Zhang, Yuanyuan Zhao, Jianxi Huang

**Affiliations:** ^1^ College of Land Science and Technology, China Agricultural University, Beijing, China; ^2^ Key Laboratory of Remote Sensing for Agri-Hazards, Ministry of Agriculture and Rural Affairs, Beijing, China

**Keywords:** maize inbred lines, WOFOST, data assimilation, Sentinel-2, EnKF

## Abstract

Maize is the most widely planted food crop in China, and maize inbred lines, as the basis of maize genetic breeding and seed breeding, have a significant impact on China’s seed security and food safety. Satellite remote sensing technology has been widely used for growth monitoring and yield estimation of various crops, but it is still doubtful whether the existing remote sensing monitoring means can distinguish the growth difference between maize inbred lines and hybrids and accurately estimate the yield of maize inbred lines. This paper explores a method for estimating the yield of maize inbred lines based on the assimilation of crop models and remote sensing data, initially solves the problem. At first, this paper analyzed the WOFOST(World Food Studies)model parameter sensitivity and used the MCMC(Markov Chain Monte Carlo) method to calibrate the sensitive parameters to obtain the parameter set of maize inbred lines differing from common hybrid maize; then the vegetation indices were selected to establish an empirical model with the measured LAI(Leaf Area Index) at three key development stages to obtain the remotely sensed estimated LAI; finally, the yield of maize inbred lines in the study area was estimated and mapped pixel by pixel using the EnKF(Ensemble Kalman Filter) data assimilation algorithm. Also, this paper compares a method of assimilation by setting a single parameter. Instead of the WOFOST parameter optimization process, a parameter representing the growth weakness of the inbred lines was set in WOFOST to distinguish the inbred lines from the hybrids. The results showed that the yield estimated by the two methods compared with the field measured yield data had R^2^: 0.56 and 0.18, and RMSE: 684.90 Kg/Ha and 949.95 Kg/Ha, respectively, which proved that the crop growth model of maize inbred lines established in this study combined with the data assimilation method could initially achieve the growth monitoring and yield estimation of maize inbred lines.

## Introduction

Agricultural information technology and intelligence have become the global trend of agricultural development, and the seed industry, as the “chip” of agriculture, has a vital strategic position in solving the world food problem and protecting national food security. In recent years, the Internet of Things, UAV(Unmanned Aerial Vehicle) remote sensing, satellite remote sensing technology and other high-tech information technology has also been successfully used in breeding, seed breeding, variety promotion and other aspects (Sishodia et al., 2020; Weiss et al., 2020). Among the world’s major food crops, maize cultivation area accounts for about 1/10 of the global cultivated area, while in China, the planted area of maize reached about 9 billion hectares, accounting for about 1/3 of the total cultivated area in the country (Ren et al., 2020; Zhang et al., 2020). At the same time, about 60 million hectares of seed production fields are needed each year to meet the needs of more than 9 billion hectares of common field corn with seeds in China (Zhong et al., 2020). As the basis for the development of the maize seed industry, maize inbred lines are clearly distinguished from maize hybrids in terms of their growth and physicochemical parameters. In recent years, some scholars have gradually applied remote sensing technology to maize breeding and seed production, and some studies have used UAV remote sensing technology to monitor maize phenotypes and assist in maize breeding (Hui et al., 2018; Zhu et al., 2020); In the seed production segment, some studies combined multivariate remote sensing data to identify maize seed production fields and distinguish maize field production scenarios from maize seed production fields (Zhang et al., 2020), results indicate that maize inbreds and hybrids need to be differentiated when monitored using remote sensing, and that the two differ significantly in remote sensing spectral characteristics and textural features (Ren et al., 2020). Therefore, this study focuses on the remote sensing method for maize inbred line yield estimation, which can quickly realize a more accurate and spatially continuous maize seed yield estimation and contribute to seed production and food security. For example, the method proposed in this study can help seed enterprises to calculate the production scale and estimate the economic benefits, and governmental organizations can also grasp the regional seed production situation based on the method proposed in this study, so as to adjust the planting policy in a timely manner to ensure food security.

In crop yield estimation, the traditional method mainly relies on field yield measurement and sampling statistics, but this method has poor timeliness, high cost and low accuracy. The remote sensing data has been widely used in the field of crop yield estimation with its advantages of high timeliness, large scope and rich information content (Bégué et al., 2018; Benami et al., 2021). Spectral information contained in each band of remote sensing data and vegetation indices calculated based on spectral reflectance (Giovos et al., 2021; Hu et al., 2021) can reflect the crop growth condition and has high accuracy. There are two main approaches to combining remote sensing data for crop yield estimation. The first one uses a crop growth model to simulate the entire growth process of the crop to obtain yield estimations, remotely sensed data are often used as observations with spatial continuity to correct model-simulated tracks, This approach is based on the principles of yield formation and is highly mechanistic. However, the algorithm is complex, computationally inefficient, and difficult to operate on a large scale (Wu et al., 2021; Cui et al., 2022; Ji et al., 2022). The second one is based on the principle of statistics, selecting remote sensing indicators with strong correlated with yield, and using simple regression, machine learning, deep learning and other statistical methods to establish regression models, so as to estimate crop yield. This way is data-driven and computationally fast, but requires a large amount of data for model training and does not have strong spatial generalization capability. Both methods have a large number of applications (Maimaitijiang et al., 2020; Tian et al., 2021). The paper focuses on exploring the differences in growth simulation between maize inbred lines and field maize mechanistically, and the study area is small, so the method of crop model and remote sensing data assimilaWtion is chosen.

When using the assimilation method for yield estimation, various types of data such as meteorological, soil data, and agromanagement are first entered into the crop model (WOFOST, DSSAT, APISIM, AquaCrop, etc). Crop growth is simulated, while leaf area index(LAI), soil moisture(SM) and chlorophyll fluorescence are introduced as assimilation variables for remote sensing observations for assimilation and correction of crop model simulation results. In assimilation systems, two issues, parameter sensitivity and parameter calibration of crop models, data assimilation strategies and algorithms, have been the focus of the researches. Some studies (Attia et al., 2021; Pereira et al., 2021) analyze crop parameter sensitivity by sensitivity analysis methods such as Sobol, Fourier amplitude sensitivity test(FAST), and extended Fourier amplitude sensitivity test(EFAST), followed by calibration of crop model sensitive parameters using parameter optimization methods such as Markov chain Monte Carlo(MCMC), differential evolutionary Markov chain(DEMC), simulated annealing(SA), and robust parameter estimation(ROPE), It enables the model to achieve high accuracy spatial migration with the help of a small amount of field observation data (Wu et al., 2022a; Wu et al., 2022b; Zhuo et al., 2022b). However, most of these studies were conducted for the use of the model across regions with parameter correction (Attia et al., 2021), while only few studies were conducted for parameter optimization aiming at different varieties of the same crop, inbred lines and hybrids (Tewes et al., 2020). In the study of assimilation strategies and algorithms, parameter optimization methods based on cost functions and ensemble filtering methods based on estimation theory are the two main types of data assimilation methods. A systematic review of the assimilation strategies of remote sensing and crop models has been carried out by many scholars (Jin et al., 2018; Karthikeyan et al., 2020). The parameter optimization method iteratively adjusts the parameters or initial conditions in the crop model to minimize the difference between the remotely sensed observations and the model simulated values for the purpose of optimizing the crop model, simplex search algorithm, maximum likelihood method, composite hybrid evolutionary algorithm (SCE-UA), Powell conjugate direction method, particle swarm algorithm (PSO), genetic algorithm (GA), simulated annealing method (SA) and other methods are applied (Huang et al., 2015; Huang et al., 2016; Gaso et al., 2021); The construction of the cost function is in the form of root mean square error(RMSE), least squares, three-dimensional variational (3DVar), four-dimensional variational (4DVar), etc. (Huang et al., 2020; Wu et al., 2021). Sequential filtering allows the state variables simulated by the model to be continuously updated to optimal forecast values, and is a data assimilation method that is time-continuous and can be applied to real-time simulations. The commonly used sequential filtering algorithms are extended kalman filter (EKF), ensemble kalman filter (EnKF) and particle filter (PF) algorithms (Kang and Özdoğan, 2019). ENKF is the most representative and widely used sequential assimilation algorithm, which has been proved to be reliable in crop modeling and remote sensing data assimilation studies (Huang et al., 2019), so ENKF algorithm is chosen in this paper.

In summary, this study focuses on resolving the following questions:

(1) It is still questionable whether the research method of crop model assimilation with remote sensing data can simulate the growth differences between crop inbred lines and hybrids and whether it can accurately estimate the yield of maize inbred lines.
(2) In studies of WOFOST models simulating maize growth, the models are usually calibrated to distinguish between different regions, different growing environments, and other issues, and few studies have been conducted on the calibration between inbred lines and hybrids of the same crop. There is also a lack of WOFOST parameter sets describing the growth of maize inbred lines.
(3) There have been many studies on the assimilation of crop models with remote sensing data using low and medium resolution (MODIS, etc.) pixels as assimilation units, but the accuracy when using high spatial resolution remote sensing images for fine crop monitoring and yield estimation, as well as the scale effects between data when validating them, are still open to discussion.

Therefore, we used sentinel-2 satellite remote sensing data assimilated with the WOFOST model to estimate the yield of maize inbred lines and validated using field yield measurement data to answer the above scientific questions initially.

## Materials and methods

### Study area

This study was conducted in a maize seed production base within Ganzhou District, Zhangye City, Gansu Province, People’s Republic of China. Ganzhou District is the largest hybrid maize seed production county in China, with a stable seed production area of more than 9 million hectares and an annual output of more than 320 million Kg, accounting for one-third of the national maize seed consumption, and the maize seed production situation in Ganzhou District is closely related to the national seed security and food security. Ganzhou District is located in the central part of Gansu Province (between 100°6′-100°52′East and 38°32′-39°24′North), Ganzhou District is a typical temperate continental climate, with an annual average temperature of 6-8°C and an average daily temperature difference of 13.4°C throughout the maize reproductive period -18.2°C between; ≥0°C accumulation temperature 2734°C, accumulation temperature over 10°C is 2140°C,; frost-free period 112-165 days; agricultural area altitude 1200-2500m, annual sunshine hours 3000-3600 hours, total annual solar radiation 147.99cal/m2.

**Figure 1:**
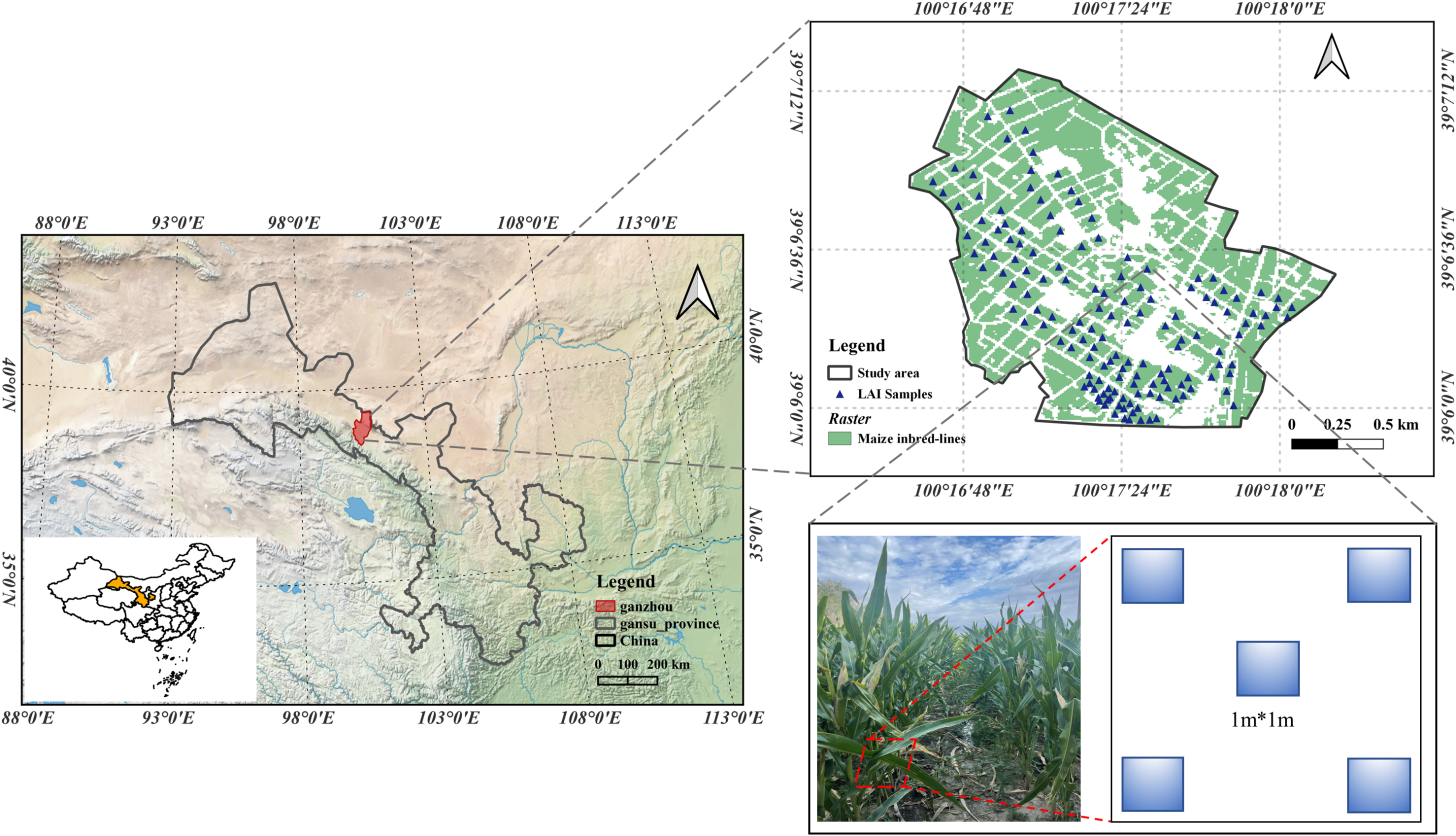
Study area, field observations distribution and strategy of LAI sampling.

The study area covers about 49,500 hectares, all planted with maize self-crosses for maize seed production, and using a threshold segmentation method to extract sentinel-2 pixels which strictly contain maize. The maize self-incompatible lines in the study area were planted at the end of April and harvested at the end of September, all under mulch drip irrigation with good water and fertilizer conditions. The three key fertility processes selected for this study were: nodulation (mid-June to end-June,2021), tasseling (end-July to early August,2021), and lactation (early to mid-September,2021). The spatial distribution of measurement sample points for field LAI measurements conducted at each of the three fertility stages is also shown in **Figure 1**:

### Data collection

A total of five datasets were used in this study, including one remote sensing dataset, two station observations and two field measurements:

1)Remote sensing data, Sentinel-2 Level-2A images of three key fertility stages of maize inbred lines with a spatial resolution of 10m, were clipped and band synthesized to invert the leaf area index in the study area.

2)Meteorological data, the meteorological data used in this paper is the daily value dataset (V3.0) of climate information from Chinese ground-based international exchange stations(dataset and its reference are in the data availability statement), which contains daily value data from 824 basic meteorological stations in China, covering parameters such as air pressure, air temperature, precipitation, evaporation, relative humidity, wind direction, wind speed, sunshine hours, etc. As required by the model inputs, (n.d.)the meteorological data used in this study include six observed quantities: daily maximum and minimum temperature, air pressure, wind speed, precipitation, and radiation. Among them, radiation is calculated from hours of bright sunshine, and the calculation method is based on the method proposed by FAO (Allen et al., 1998), The specific algorithm is as follows:


(1) $$R_{S}=\left(a_{S}+b_{S}\frac{n}{N}\right)R_{a}$$

(2) $$R_{a}=\frac{24\left(60\right)}{\pi }G_{SC}d_{r}\left[\omega_{S}\sin\left(\phi \right)\sin\left(\delta \right)+\cos\left(\phi \right)\cos\left(\delta \right)\sin\left(\omega_{S}\right)\right]$$

(3) $$\omega_{S}=\arccos\left[-\tan\left(\phi \right)\tan\left(\delta \right)\right]$$

$R_{S}$ is Atmospheric upper bound incident radiation, n is hours of bright sunshine, N is hours of possible sunshine, $a_{S}$ and $b_{S}$ are Empirical constants, taken as 0.25 and 0.5 respectively, $G_{SC}$ is Solar Constant, $d_{r}$ is the relative distance between the sun and the earth, $\omega_{S}$ is the solar time angle, ϕ is latitude, δ is the solar declination.

3)For soil data, soil property data were obtained from the Food and Agriculture Organization of the United Nations (FAO) soil dataset with a spatial resolution of 5 min, including parameters such as permeability, field water holding capacity, and wilting point water content for different soil textures, while soil moisture data were also obtained from the meteorological stations mentioned above.

**Figure 2:**
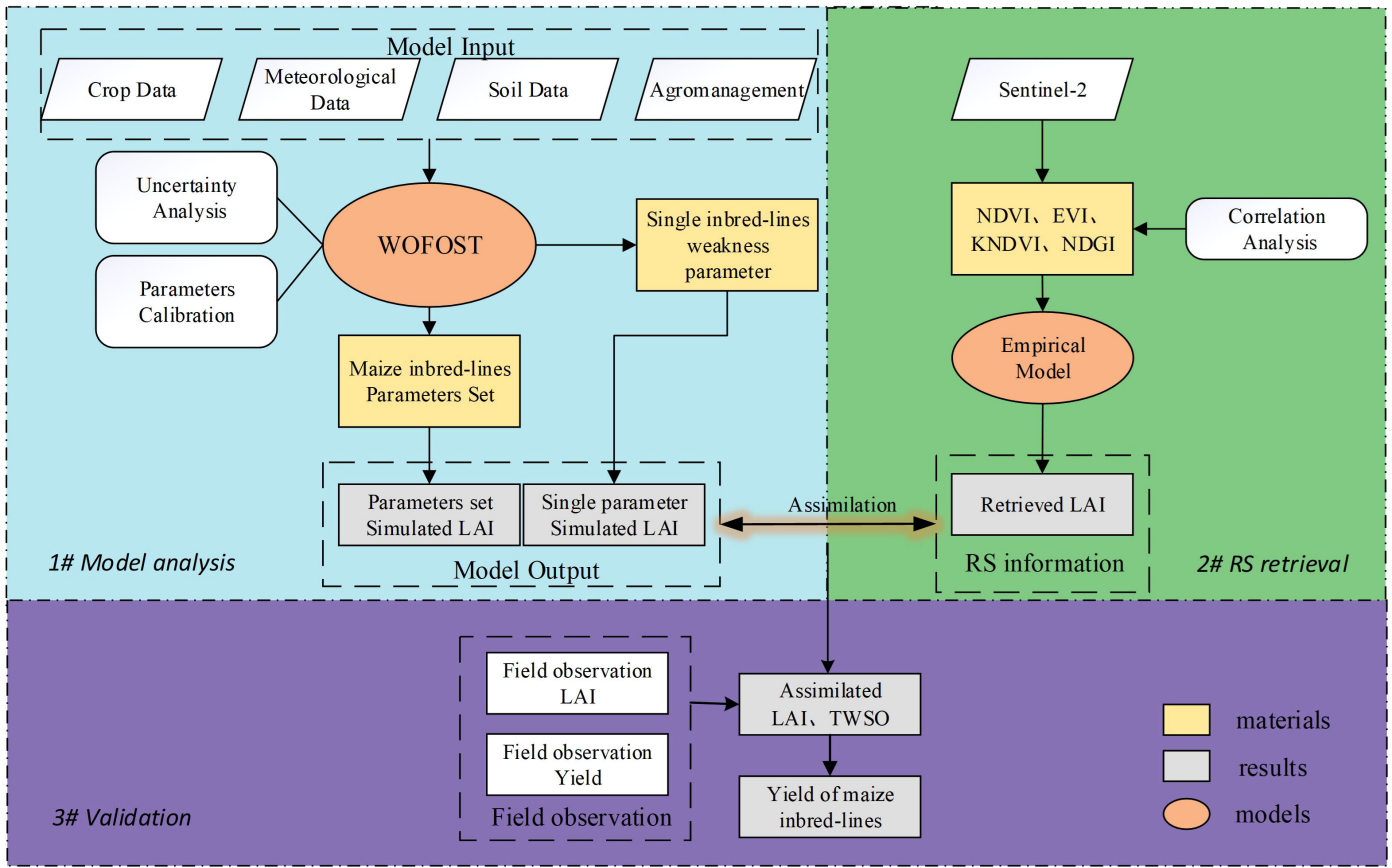
Overview of the research.

4)The field measurements of LAI data were carried out three times at the jointing stage (June 22, 2021), tasseling (August 6, 2021), and milky (September 3, 2021) stages of the maize inbred lines, and the sample points were distributed as shown in **Figure 2**. Two rows of maize inbred lines (parent or female) were randomly selected for the five-point sampling method, and then the leaf area index of the sample points was obtained by taking the average value, and the measurement was repeated three times. The sample points with the standard deviation of the three measurements less than 25% were selected as valid measurement points, and the actual LAI data in the study area were finally obtained.

5)Phenological information and field yield measurement data were collected from the maize seed production base agronomist records, and yield measurements were obtained at the maturity of the maize inbred lines (September 10, 2021) using standard agronomic yield measurement methods (calculated from number of ears, number of grains, and thousand grain weight).

### Methods

#### Overview

This study consists of several steps, as shown in the figure:

The first part is the model analysis, we input the required data for the model, run the model, and at the same time, use the Sobol global sensitivity analysis method to analyze the crop parameters of the WOFOST model and select the parameters that need to be calibrated; then use the MCMC parameter calibration method to calibrate the sensitive parameters to obtain the parameter set of maize inbred lines, and at the same time, set an independent parameter in the model to represent the inbred lines In the assimilation process, we tried to optimize this parameter to explore whether the difference in growth between the self and hybrid could be described by a single parameter; finally, we used the calibrated parameter set for simulation to obtain the LAI of the inbred lines. The second part is the remote sensing estimation of LAI. Four vegetation indices were selected to establish linear regression models at each of the three fertility stages for estimation of the LAI of maize inbred lines in the study area. the third part is data assimilation and validation, using LAI as the assimilation variable to assimilate the simulation results of the above two parts, and finally to obtain the yield estimation results of maize inbred lines combining remote sensing observations and crop models, and to evaluate the simulation accuracy using ground observations for validation.

#### Estimation method of LAI

When using the data assimilation algorithm for analysis, the first step is to obtain the remote sensing estimations of the LAI of maize inbred lines on each pixel in the study area. For leaf area index estimation of a single crop in a small area, the literature (Parker, 2020; Qiao et al., 2020) shows that empirical models have the advantages of high computational efficiency, small sample requirement, and high simulation accuracy compared to more complex models such as machine learning or process models, and estimation of small areas can also avoid the disadvantages of poor robustness of empirical models. NDVI, as the most commonly used indicator for vegetation remote sensing, is usually chosen as the backbone for LAI estimation (Zeng et al., 2022). EVI can effectively address the saturation benefits of high vegetation cover compared to NDVI, and some studies have used EVI as the main feature for remote sensing identification of seed production maize, and the results proved that EVI has a strong correlation with maize inbred lines’ LAI (Ren et al., 2020). NDGI is used to characterize the information of vegetation greenness, and the correlation with the relationship with biomass in the early stage of crop growth is very high (Cao et al., 2020). kNDVI was proposed in 2021 as a new type of vegetation index obtained by treating NDVI with a kernel function. The correlation of kNDVI with LAI and biomass is greatly improved compared to existing vegetation indices and has been validated at the global scale (Camps-Valls et al., 2021).

Therefore, in this study, four vegetation indices closely related to the leaf area index were selected. We then used the four vegetation indices calculated from Sentinel-2 satellite imagery to do a one-dimensional linear regression with the field-measured LAI data at each of the three fertility periods of the maize inbred lines to select the optimal estimation model. The four vegetation indices and their calculation methods are shown in **Table 1**.

**Table 1:** Equations of vegetation indices.

VIs	Equation	Full name	Reference
NDVI	NDVI=(Rnir−Rred)/(Rnir+Rred) (4)	Normalized Difference Vegetation Index	(Tucker, 1979)
EVI	EVI=2.5·(Rnir−Rred)/(Rnir+6·Rred−7.5·Rblue+1) (5)	Enhanced Vegetation Index	(Huete et al., 2002)
NDGI	$NDGI=\left(R_{green}-R_{red}\right)/\left(R_{green}+R_{red}\right)$ (6)	Normalized Difference Greenness Index	(Nedkov, 2017)
kNDVI	$kNDVI=\tanh\left(NDVI^{2}\right)$ (7)	Kernel Normalized Difference Vegetation Index	(Camps-Valls et al., 2021)

#### Model description

The WOFOST model, jointly developed by Wageningen University and the World Food Research Center in the Netherlands, is capable of dynamically simulating the growth of crops under specific climatic and soil conditions over the reproductive period in daily steps, and the WOFOST model is one of the most commonly used crop growth models in recent years in the field of remotely sensed crop growth monitoring and yield estimation (Zhuo et al., 2022a). The model is driven by day-by-day meteorological data to explain the effects of light and heat conditions, soil conditions, and crop varieties on crop growth by simulating crop respiration, photosynthesis, transpiration, material partitioning, and leaf senescence, and ultimately to simulate crop growth, and to support the simulation of three crop growth conditions: potential growth patterns, water stress, and nutrient stress. The model is ultimately able to simulate the total dry matter weight of crop storage organs, which is then converted to obtain crop yields by converting the standard water content. The reason for choosing the WOFOST model in this study is that the WOFOST model uses more parameters to simulate the process of leaf development as well as yield formation compared to Aquacrop, Dssat, etc., and can better explain the differences between maize autogamy and hybrids based on the differences in model parameters (De Wit et al., 2019). Moreover, WOFOST is the most widely used in the study of assimilation of remote sensing data and crop models for yield estimation (Huang et al., 2019).

This study uses the WOFOST model in the potential model, which requires four sets of data as inputs: meteorological data, soil data, field management, and crop variety parameters. The meteorological parameters include daily maximum and minimum temperature, wind speed, precipitation, and sunshine duration, etc. The soil data include field water holding capacity, saturation water content, and wilting coefficient, etc. The field management part needs to be recorded in the field to obtain them, and the crop variety parameters are the most important part and the focus of this study. The Python Crop Simulation Environment (PCSE) framework provides an environment for operating the WOFOST crop growth model, and the code for the data assimilation algorithm was written in the Python language in the Windows 10 operating system.

#### WOFOST parameter sensitivity analysis method

The Sobol algorithm is a global parameter sensitivity analysis method based on the variance decomposition principle, which decomposes the total variance of the objective function into the variance of each individual variable and the interaction variance of each variable, and uses it to calculate the first-order sensitivity parameters and interaction sensitivity parameters of the parameters (Zhang et al., 2021).The advantage of the Sobol algorithm as a common sensitivity analysis algorithm for crop model analysis is that it can calculate global sensitivity and can analyze the interaction between parameters, and the disadvantage is that it is computationally intensive. The specific algorithm principle is as follows.


(8) $$f\left(x\right)=f_{0}+\sum_{i}f_{i}\left(x_{i}\right)+\sum_{i<j}f_{ij}\left(x_{i},x_{j}\right)+\cdots +\sum_{i<j}f_{1,2,\cdots ,n}\left(x_{1},x_{2},\cdots ,x_{n}\right)$$

Where: f(x) is integrable and the time variable x conforms to a uniform distribution in [0, 1],if f(x) satisfies:


(9) $$\int_{0}^{1}f_{i_{1}\cdots i_{j}}\left(x_{i},\cdots ,x_{i_{j}}\right)dx_{k}=0$$

Variance function: f(x) can be decomposed into single-parameter variance with multi-parameter interactions.


(10) $$\sum_{i}\frac{D_{i}}{D}+\sum_{i<j}\frac{D_{ij}}{D}+\cdots +\frac{D_{1,2,\cdots ,n}}{D}=1$$

Where: D represents the total variance of the function; $\;D_{i}$ represents $x_{i}$ generating variance; $\;D_{ij}$ represents Interaction produces variance over $x_{i}$ and $x_{j}$ ; $\;D_{1,2,\cdots ,n}$ represents n parameters acting together to produce the variance. Thus, parameter first-order sensitivity $SC_{i}$ , interaction sensitivity $SC_{ij}$ , total sensitivity $SC_{Ti}$ can be represented as:


(11) $$SC_{i}=D_{i}/D$$

(12) $$SC_{ij}=D_{ij}/D$$

(13) $$SC_{Ti}=1-D_{\sim i}/D$$

#### WOFOST model parameter optimization method

MCMC (Monte Carlo Markov Chain) is a parameter calibration method based on Bayesian theory, which has been widely used in the parameter calibration of various crop growth models as well as leaf spectral models. Bayesian theory can calculate the posterior distribution of the model parameters based on the observed values corresponding to the model output variables, combined with the prior distribution of the model input parameters, and the principle of the algorithm is shown in Equation 11. In contrast, the MCMC method is a Markov chain introduced into the Monte Carlo stochastic process, which can achieve a sampling distribution that changes dynamically according to the simulation results and converges the Markov chain to achieve a steady-state distribution. The specific principle of the MCMC algorithm is shown in paper (Andrieu et al., 2003).


(14) $$p\left(\theta /,y\right)=\frac{f\left(y/\theta \right)g\left(\theta \right)}{\int f\left(y/\theta \right)g\left(\theta \right)d\theta }$$

where: θ represents WOFOST model parameters, y represents model result (Only LAI in this study), p(θ,y)  represents the posterior probability density function of the parameters, f(y,θ)  represents the observed value likelihood function, g(θ) represents the prior distribution of the parameters

#### Data assimilation method

The ensemble Kalman filter (EnKF) is a sequential assimilation method, and the principle of its algorithm can be explained in the context of this study as follows: the trajectory of the model simulation is continuously adjusted by incorporating external remote sensing observations in the simulation framework of the crop model, and the simulation error is reduced. EnKF assumes that the observations and the model are Gaussian distributed, and has the ability to handle nonlinear observations by means of ensemble forecasting, and is the most promising method for assimilating yield estimation It is the most promising sequential assimilation method in the study (Evensen, 2003). The specific steps of its algorithm are as follows:


(15) $$A_{k}^{f}=M\left(A_{k-1}^{\alpha }\right)$$

(16) $$A_{k}^{\alpha }=A_{k}^{f}+P_{k}H^{T}{\left(HP_{k}H^{T}+R_{k}\right)}^{-1}\left(D_{k}-HA_{k}^{f}\right)$$

Among them: $A_{k}^{f}$ is the forecast matrix for the set of state variables at moment k, $A_{k}^{\alpha }$ is the analysis matrix of the set of state variables at moment k, $P_{k}$ is the covariance matrix of the forecast matrix, $R_{k}$ is the covariance matrix of the observation matrix, H is the observation operator, $D_{k}$ is the observation matrix, M is the state transformation equation.

In this study, the crop model was first run using default parameters to simulate maize growth, and LAI was selected as the assimilation variable, and a Monte Carlo method was used to perturb LAI and set up to generate forecast ensembles at three periods of maize jointing stage, tasseling, and milky stage. The standard deviation of the simulated LAI values was 0.20 and the standard deviation of the observed LAI values was 0.25 based on the error between the measured data and the model simulation results. samples were then extracted from the sampling obeying Gaussian distribution to generate the forecast ensemble. The EnKF algorithm was run iteratively at three key fertility stages to drive the model simulation to the maturity of the maize inbred lines, and finally the assimilated simulation results were obtained.

#### Method of establishing growth weakness parameter of maize inbred lines

In addition to the optimization of WOFOST sensitive parameters using the parameter calibration method, this study also proposes a method to differentiate maize inbred lines from hybrids: a parameter used to describe the growth weakness of inbred lines is added to the crop parameter module of the WOFOST model, and according to the study (Lanza et al., 1997; Gunjaca et al., 2008), inbred lines of the same crop usually grow 30 to 80 percent as long as hybrids, and the gramineous crops are usually about 70 percent as long. Therefore, the initial value of this coefficient is set to 0.7 and the upper and lower bounds of the parameter are set to [0.3,0.8]. The purpose of this is that: It would greatly reduce the Computation of parameter optimization if only a single coefficient could be used to simulate the growth differences between maize inbred lines and hybrids in the EnKF algorithm assimilation process.

## Results and discussion

### LAI remote sensing estimation results

**Figure 3:**
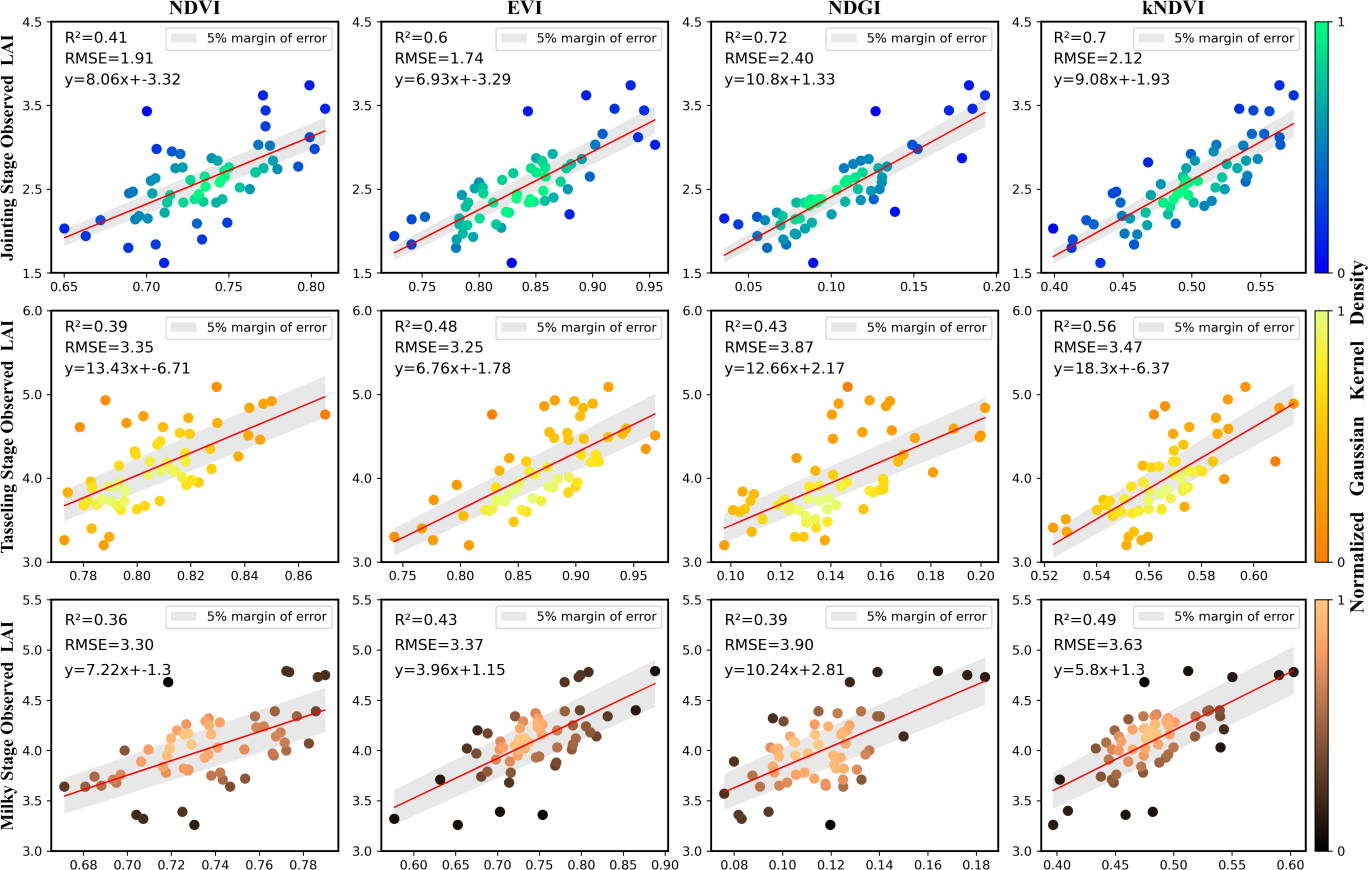
Validation of the models using 30% independent samples in three development stages.

In the process of data assimilation of LAI as a state variable, it is first necessary to achieve spatially continuous remote sensing estimation of LAI in the study area. In this study, four vegetation indices (NDVI, EVI, NDGI, and kNDVI) calculated from Sentinel-2 satellite images were used to establish linear regression models at three growth stages of maize inbred lines, namely, the jointing, tasseling, and milky stages, respectively, while the samples were divided in order to evaluate the stability and accuracy of the models, of which 70% were used to establish the models and 30% were used to validate the models, and the validation results are shown in the following **Figure 3**.

The results showed that the correlation between the four vegetation indices and the leaf area index of the maize inbred was highly significant (p<0.01) at each growth period, which also proved the feasibility of the linear regression estimation of LAI using vegetation indices. As shown in the **Table 2**, the models with the highest accuracy were selected to invert the LAI of maize inbred lines in three periods. NDGI had the highest accuracy at the jointing stage, with R^2^ and RMSE of 0.72 and 2.4 m^2^/m^2^, respectively, and the other three vegetation indices were also estimated with high accuracy. In the tasseling stage, kNDVI had the highest estimation accuracy, with R^2^ and RMSE of 0.56 and 3.47 m^2^/m^2^, respectively, and it could be seen that the estimation accuracy of the model decreased in the tasseling stage compared with the jointing stage. In the milky period, kNDVI also had the highest accuracy, with R^2^ and RMSE of 0.49 and 3.63 m^2^/m^2^, respectively, and the accuracy of the model for all vegetation indices decreased further compared with the previous two stages.

**Table 2:** Empirical models and model evaluations.

Growth stage	Vegetation index	Model	Model accuracy	
			R^2^	RMSE
Jointing Stage	NDGI	y=10.8x+1.3	0.72	2.40 m^2^/m^2^
Tasseling Stage	kNDVI	y=18.3x-6.3	0.56	3.47 m^2^/m^2^
Milky Stage	kNDVI	y=5.8x+1.3	0.49	3.63 m^2^/m^2^

**Figure 4:**
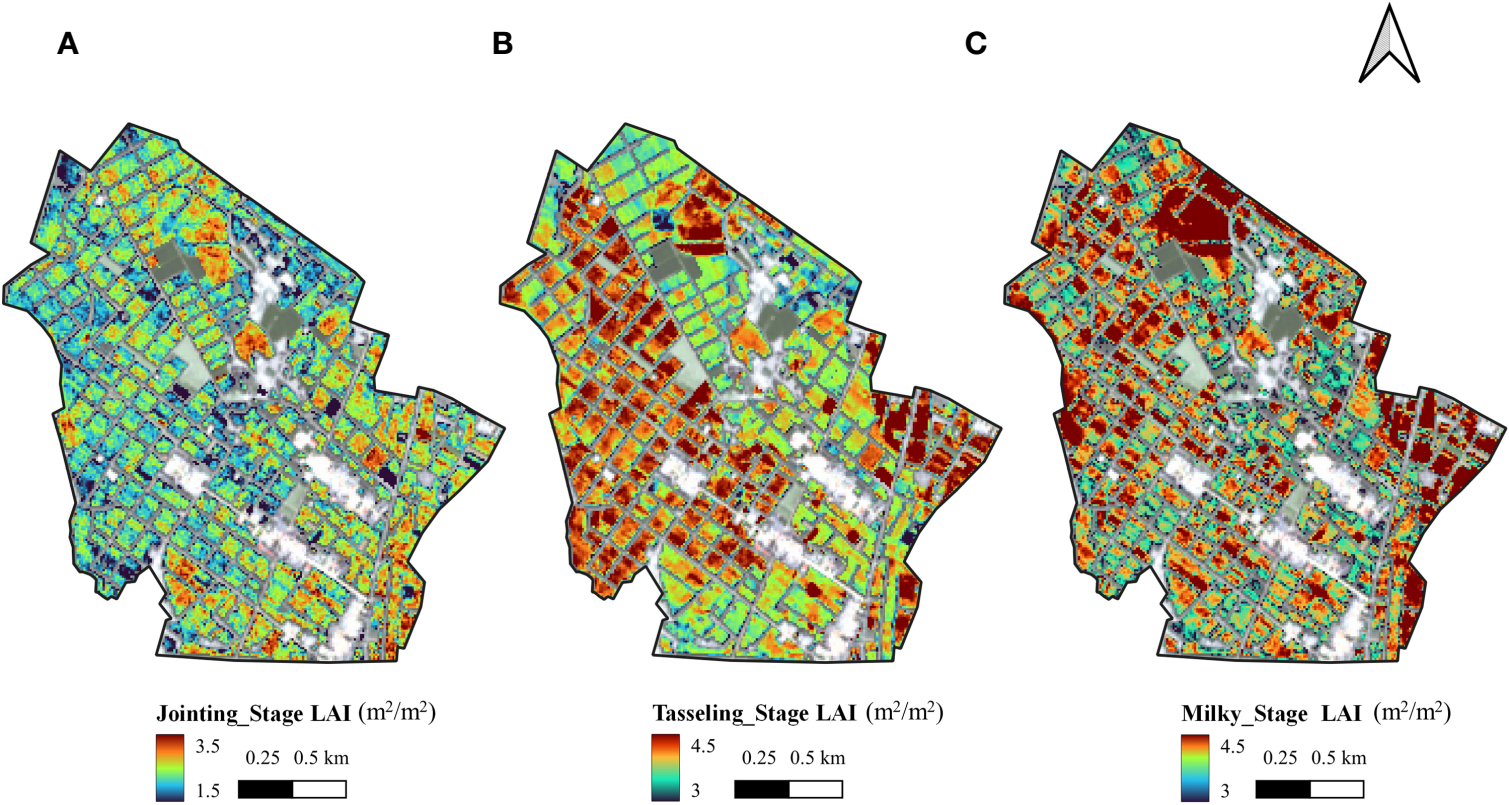
Mapping results of LAI calculated in jointing stage **(A)**, tasseling stage **(B)** and milky stage **(C)** using Sentinel-2 images.

Further analysis based on the above results showed that the maize inbred lines had a growth disadvantage compared to the hybrids, with plant height about 1 m lower and biomass about 70% lower compared to the hybrids (Lanza et al., 1997). After the jointing stage, the plant height and leaf changes of maize inbred lines were subtle and slow, so the canopy saturation phenomenon was earlier and more pronounced in the spectral response of the canopy on the phenological stage. At the jointing stage, the maize inbred lines grow rapidly and the reflectance in the green band changes significantly, so NDGI can fit the LAI well. at the tasseling and milky stages, the traditional linear vegetation index cannot invert the LAI of inbred lines well, while kNDVI can fit the canopy spectral response of the maize inbred lines better due to its introduction of the RBF kernel function (Camps-Valls et al., 2021), so it has a better estimation effect. The estimating results of the leaf area index of maize inbred lines in the study area are shown in **Figure 4**:

### Model sensitivity analysis and parameter calibration

#### Sensitivity analysis

According to the study, 36 parameters related to leaf development, photosynthesis and material partitioning in the crop parameter module of the WOFOST model were selected for model sensitivity testing, and the upper and lower limits of the parameter values were referred to the upper and lower limits of the parameters in the WOFOST model description document, as shown in **Supplementary Material**.

**Figure 5:**
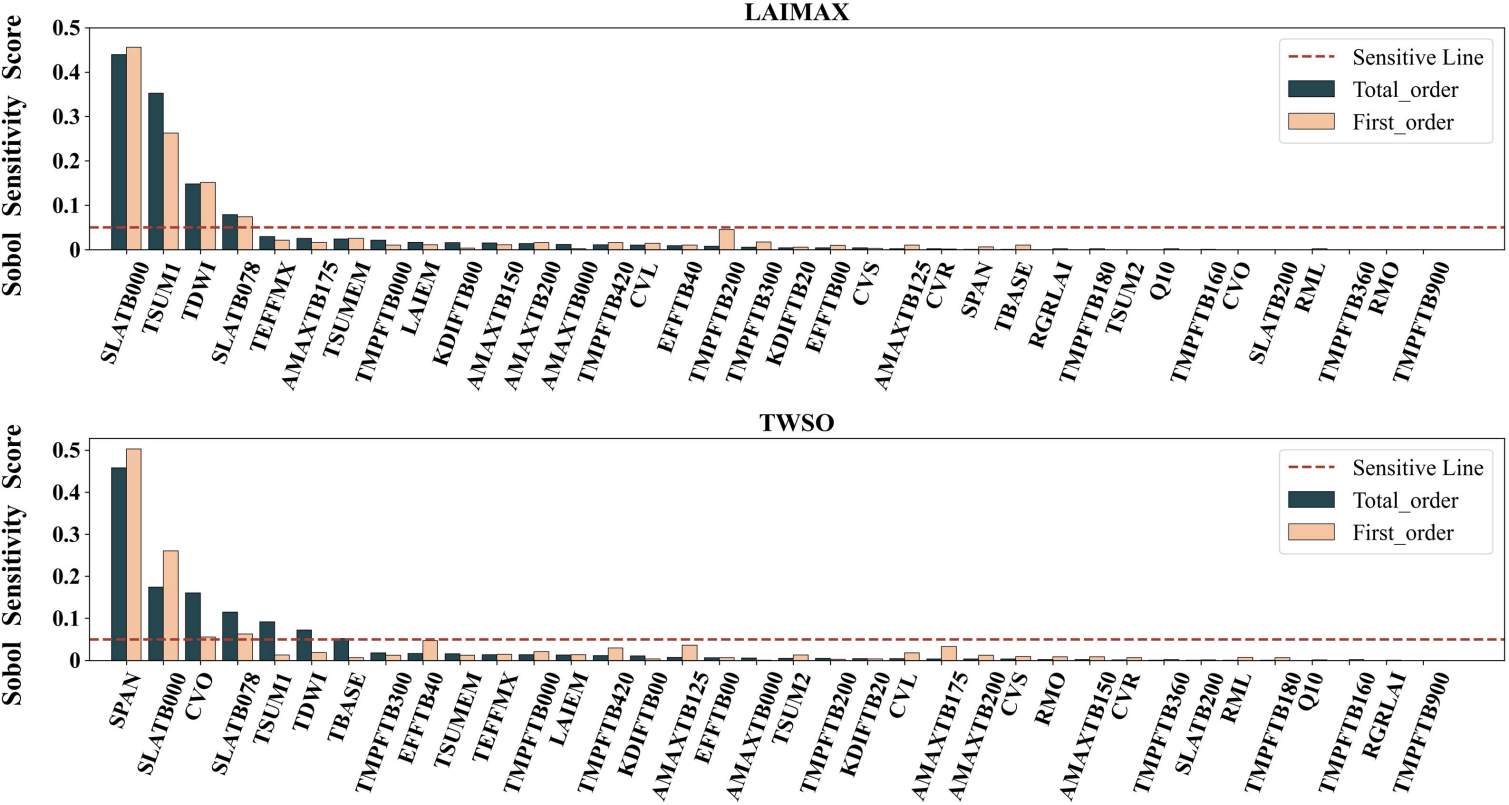
Sobol sensitivity score for two target variables: LAIMAX and TWSO.

In this study, the Sobol sensitivity analysis method based on variance decomposition was used. Since the target of the study was the yield of maize inbred lines and the assimilation variable was LAI, LAIMAX (maximum leaf area index) and TWSO (total weight of crop storage organs) of the model output variables were used as targets for sensitivity analysis, and the first-order sensitivity and global sensitivity were calculated for 36 parameters, respectively, the results are shown in **Figure 5**.

According to the literature, the parameters with Sobol sensitivity scores over 0.05 were taken as sensitive parameters, and the results showed that the first-order sensitivity and global sensitivity of three parameters, SLATB000, TSUM1, TDWI, and SLATB078, exceeded 0.05, and the first-order sensitivity of TMPFTB200 exceeded 0.05, but the global sensitivity was low, and these four parameters were sensitive to the maximum These four parameters were sensitive to the maximum leaf area index, among which the sensitivities of two parameters, SLATB000 and TSUM1, exceeded 0.2, indicating that these two parameters played a decisive role in the model simulation of maximum leaf area; while the seven parameters, SPAN, SLATB000, CVO, SLATB078, TSUM1, TDWI, and TBASE, were sensitive when the model output variable was the total weight of storage organ sensitive. The intersection of the sensitive parameters of the two output variables was taken, and a total of eight sensitive parameters were obtained, which will be used as the key to distinguish the growth simulation of maize inbred lines and hybrids, and further parameter calibration.

#### Parameter calibration

After obtaining the sensitive parameters of the WOFOST model, the sensitive parameters were calibrated by the MCMC method using the LAI of the maize inbred lines obtained from field measurements. The MCMC method was used to obtain the posterior distribution of the parameters and the uncertainty assessment of the parameters by the above-mentioned computational principles with the default parameters conforming to the normal distribution at the time of sampling. Based on previous studies, the upper and lower bounds of the parameters were determined based on a 30% float of the default values (Zhuo et al., 2022a). The calibration results are specified in **Table 3**.

**Table 3:** optimized crop parameters’ values.

Parameter name	Definition	Initial value	Optimized values	95% Confidence interval
SLATB000	specific leaf area at 0.00 at the growth period	0.0026	0.002	[0.0019,0.0021]
SLATB078	specific leaf area at 0.78 at the growth period	0.0012	0.0016	[0.00152,0.00168]
CVO	efficiency of conversion into storage org	0.671	0.63	[0.5985,0.6615]
TSUM1	temperature sum from emergence to anthesis	695	735	[698.25,771.75]
TDWI	initial total crop dry weight	50	35	[33.25,36.75]
SPAN	life span of leaves growing at 35 Celsius	33	39	[37.05,40.95]
TBASE	lower threshold temperature for ageing of leaves	10	10	[9.5,10.5]
TMPFTB200	Correction factor for the maximum CO2 assimilation rate at 20°C	1	0.8	[0.76,0.84]

Four of these parameters, SLATB000, CVO, SPAN, and TMPFTB200, hybrids are taken to be larger than the inbred lines. While three parameters, SLATB078, TSUM1 and TDWI, were taken by hybrids to be less than that of the inbred lines. As for the parameter TBASE, both of them take the same value. Among the above parameters, SLATB000, SLATB078 and SPAN are all related to the leaf development of inbred lines. According to the results of this paper: when firstly emerged, the specific leaf area of the inbred lines was smaller than that of the hybrids; before tasseling (DVS<0.78), the specific leaf area was larger than that of the hybrids; meanwhile, the leaf senescence index of the inbred lines was higher than that of the hybrids. In other words, compared with the hybrids, the leaf growth rate in the early stage was lower than that of the hybrids, but before entering the tasseling stage (DVS<0.78), the leaf growth rate was higher than that of the hybrids, and the leaf senescence of the hybrids was more rapid. Among the calibration parameters: CVO, TMPFTB200, which represent the efficiency of inbred lines in terms of material transformation and carbon assimilation, respectively. The results show that: The material transformation efficiency of the seeds of the inbred lines was lower than that of the hybrids, while the CO2 assimilation efficiency of the inbred lines was 80% of that of the inbred lines at 20°C. Finally, the parameter TSUM1 represents the accumulated temperature from emergence to tasseling, The results showed that: The inbred lines require higher accumulated temperatures from emergence to tasseling than the hybrids, which means that the hybrids will tasseling faster if they are sown at the same time under the same conditions. Some studies (Betrán et al., 2003) have compared the differences in shape between maize hybrids and inbred lines under stress and non-stress environments. The results show that: The hybrids flowered earlier, had taller plants, more spikes per plant, higher dehiscence rates, slower leaf senescence, and higher leaf chlorophyll content than the inbred lines in all environments. This also corroborates with the results of this study on the calibration of parameters. Another study (Betran et al., 2003)showed that maize inbred lines have fewer seeds and faster leaf senescence than hybrids, which is also consistent with the results of the present experiment.

In previous studies, it is clear that hybrids of maize have heterozygous advantages over inbred lines in various aspects such as plant height, biomass, yield, etc. For example, (Hisse et al., 2019) study showed that: Under the same conditions: plant and tassel height were consistently higher in the hybrids than in the siblings, showing 54% and 68% hybrid advantage. And in terms of yield, (Hisse et al., 2019) showed that: The advantage of hybrids over inbred lines in grain yield is greater at high soil N than at low soil N because the growth limitation imposed by the inbred itself reduces the nutrient demand on the inbred lines, resulting in a different response to environmental changes. More studies quantified the heterosis advantage in yield, (Dong et al., 2020) shows that the yield of the progeny was increased by 24.3% to 186.5% compared to that of the inbred lines by crossing different strains of the inbred lines. And based on the crop growth model, this paper explores the key crop parameters affecting hybrid dominance from the process, and calibrates them to obtain a parameter set that can basically simulate the growth condition of the inbred lines.

**Figure 6:**
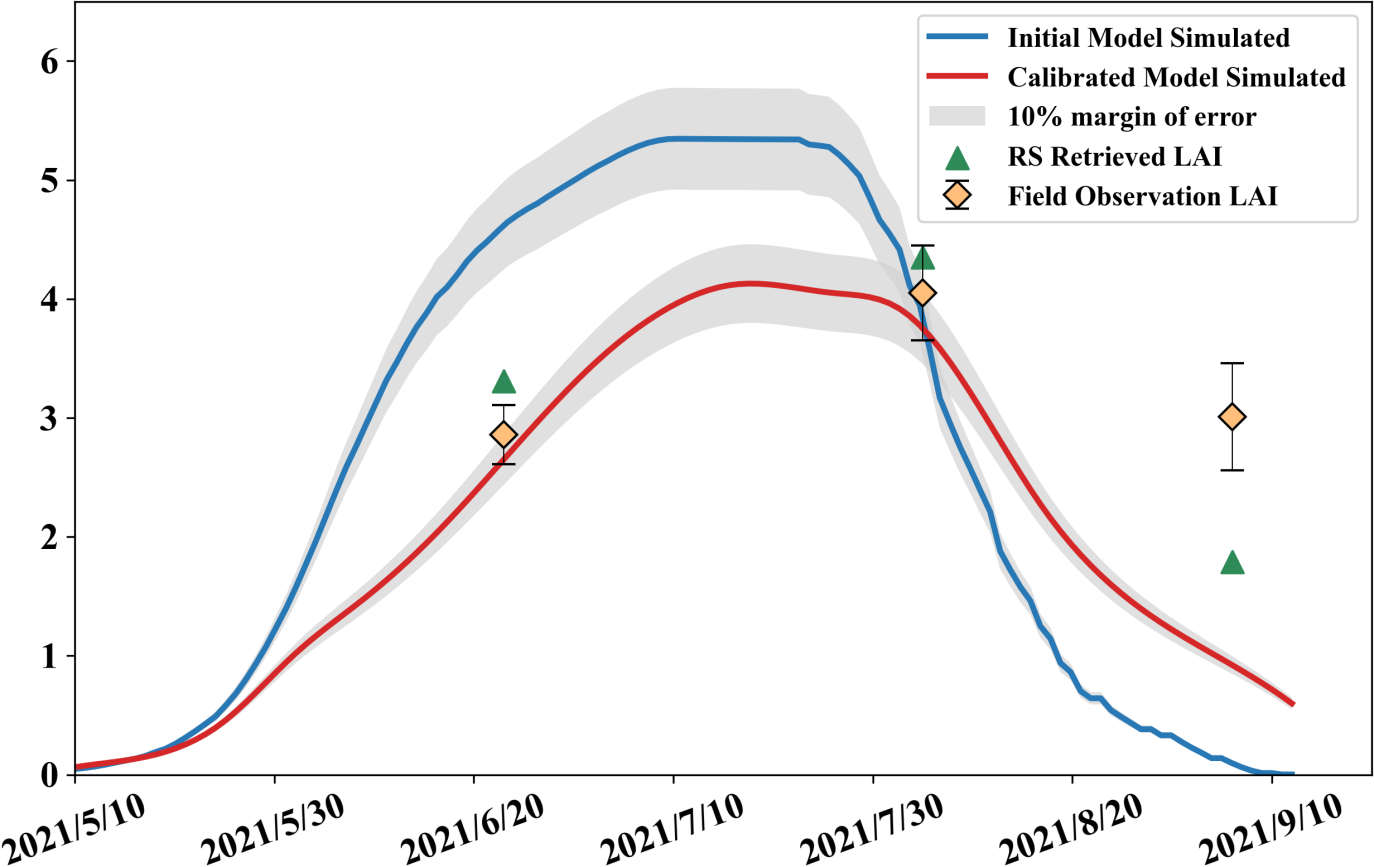
Model initially simulated LAI, optimized simulation and field observations.

Simulations were performed using the above inbred lines parameter set driving the WOFOST model, and the results are as **Figure 6**: it can be seen that the calibrated simulation results are closer to the measured values, and it has been possible to obviously simulate the growth weakness, in the last measurement, because the maize leaves have been mostly yellowed, but when using the canopy analyzer for measurement, the yellowed leaves will affect the measured values, and the crop model will only simulate the leaf area index of fresh leaves, so the measured values, remote sensing estimation, and the model simulation values are more different.

### Regional assimilation of yield tpestimation results

**Figure 7:**
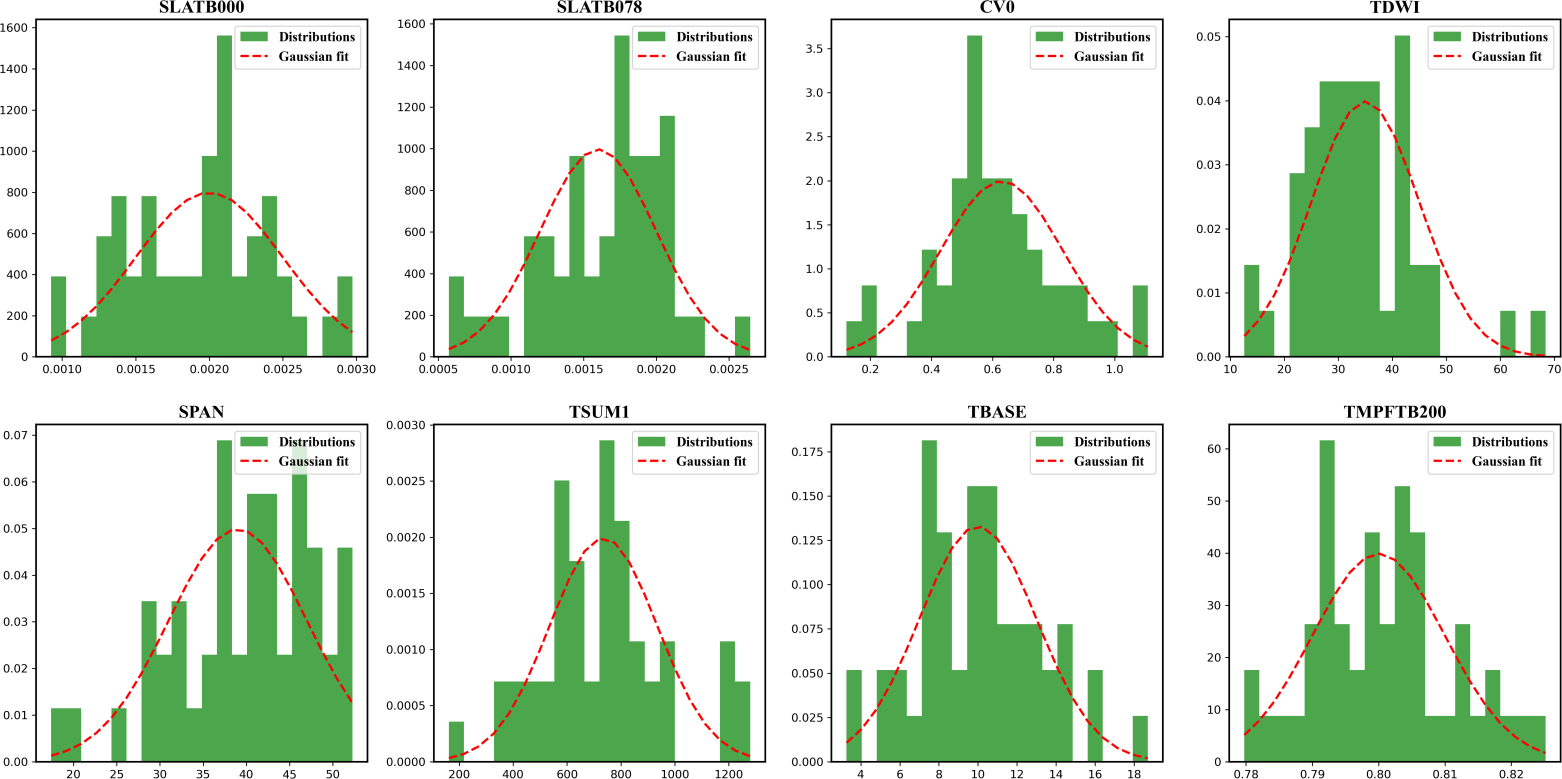
Parameters distributions and gaussian fit curves when sampling in EnKF algorithm.

After obtaining the parameter set of the calibrated inbred lines, LAI was used as the assimilation variable, and three sequential assimilations were performed using the EnKF algorithm at the jointing, tasseling, and milky stages, respectively. In the EnKF assimilation process, the above optimized parameters were considered to conform to a Gaussian distribution among the parameters, and the optimized values were used as their means, with the standard deviation determined empirically from the model, and each ensemble member from the parameter distribution was sampled, overriding its default value. The parameter sampling process for creating the ensemble is shown in **Figure 7**.

**Figure 8:**
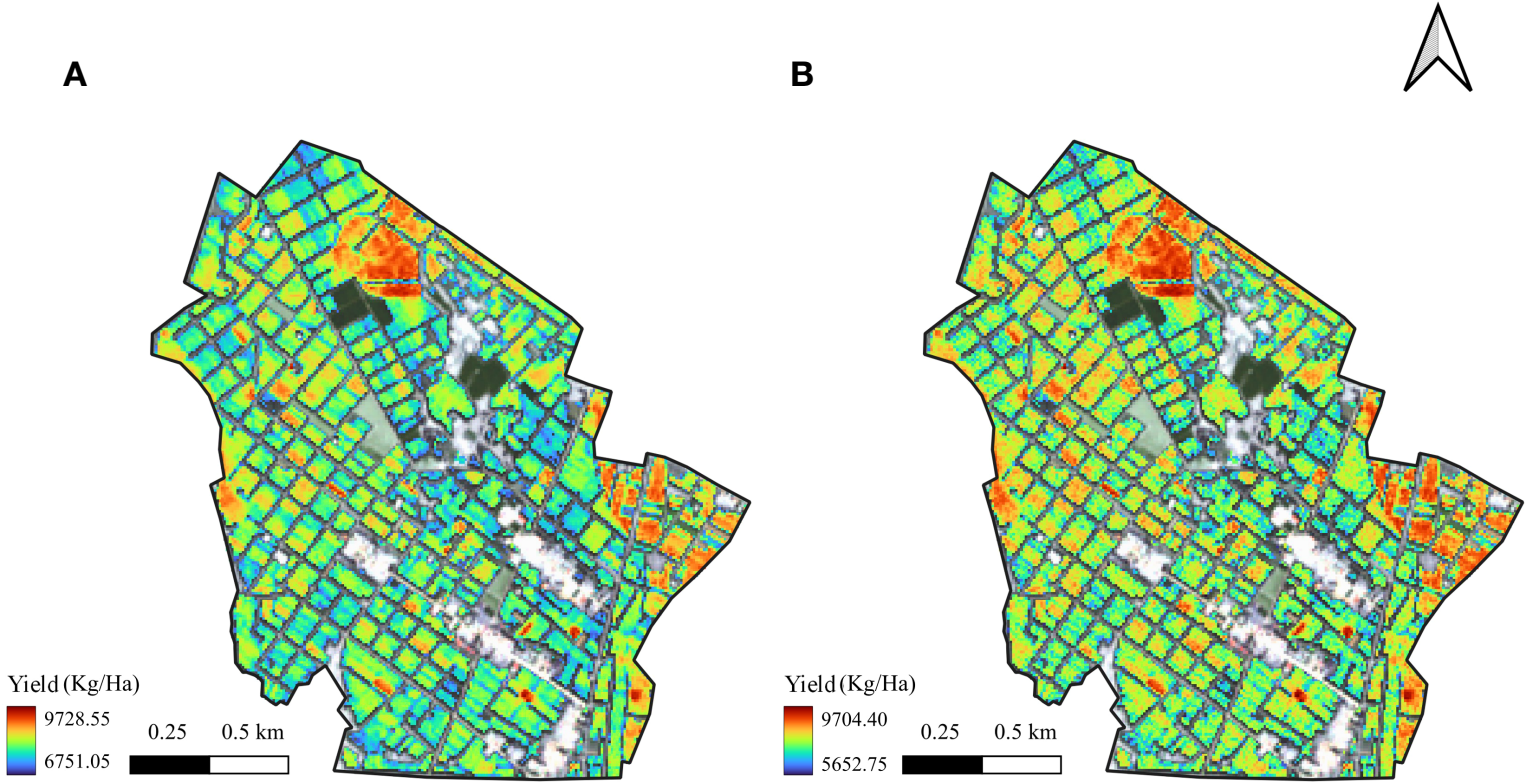
Mapping the yield estimation results in study area using two methods:**(A)** singe parameter assimilation and **(B)** parameter set assimilation.

Through the above process, the remote sensing observations were sequentially assimilated with the simulated results of the calibrated WOFOST model by means of ensemble forecasting and operated pixel by pixel to finally obtain the yield estimation results of maize inbred lines in the study area, as shown in **Figure 8**.

**Figure 9:**
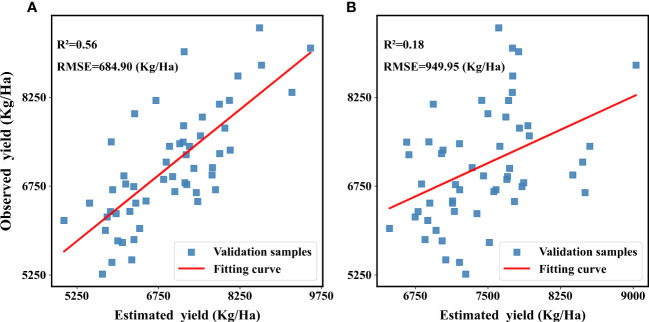
Validation the results of yield estimation of two methods:**(A)** parameter set assimilation **(B)** singe parameter assimilation.

Then, validate the yield estimating results in single parameter assimilation methods and parameter set assimilation method, as shown in **Figure 9**.

From the results, it can be seen that results of the two methods has the same spatial distributions of low and high values, but because the single parameter method optimized only one parameter, the range of the estimated results was small and the extreme condition of high and low yields could not be simulated, in comparison, the simulation of parameter set assimilation method was more robust and the simulation results were more uniformly distributed, and the simulation effect of the high and low yield cases needed to be improved. The reason for this analysis may be due to the fact that the number of calibrated parameters is not enough and there are still more parameters that have an impact on yield that have not been considered. From the validation results: single parameter assimilation method R^2 = ^0.18, RMSE=949.95 Kg/Ha; parameter set assimilation method R^2 = ^0.56, RMSE=684.90 Kg/Ha. The parameter assimilation method has higher coefficient of determination, lower root mean square error, and better estimation. In this study, the single parameter assimilation method was used in order to explore whether it is possible to simply differentiate the growth simulation process of maize inbred lines from that of hybrids by incorporating a single correction factor within the framework of the WOFOST model. However, the results proved that the single-parameter assimilation method could not accurately estimate the yield of maize inbred lines, and its accuracy was much lower than that of the traditional parameter set method. The reason for this was analyzed because in the growth differences between the inbred lines and the hybrids exceeded the limits that could be adjusted by a single parameter, so the parameter set method could better explain the differences between the two in terms of physiological processes by changing more parameters. The rationale inherent in the parameter set assimilation method is also explained in the studies (Wu et al., 2021; Ji et al., 2022).The above results show that the EnKF-based parameter set assimilation method can better estimate the yield of maize inbred lines at both single point and regional scales.

The yield estimation method for maize inbred lines proposed in this paper, although the overall values of R2 and RMSE are not too high, is the first time that the assimilation of crop model and remote sensing data is applied to inbred lines, which refines the research object from species to varieties, and provides greater possibilities for the application scenarios of crop models. Meanwhile, with reference to the method in this paper, we can get the spatially continuous yield estimation results of inbred lines on a larger scale, which improves the efficiency and accuracy of hybrid seed yield estimation, and contributes to guaranteeing the safety of the seed industry.

## Conclusion

In this paper, the WOFOST model driven by meteorological, field management, soil and crop phenology data in 2021 was used to simulate the growth of maize inbred lines and assimilated with Sentinel-2 satellite remote sensing data to successfully estimate the yield of maize inbred lines and validated with field yield measured data, using Ganzhou District, Zhangye City as the study area, and the following conclusions were obtained.

(1) Through parameter calibration, the WOFOST model can simulate the growth differences between maize inbred lines and hybrids, and can simulate key parameters such as leaf area index and above-ground biomass of maize inbred lines more accurately, which can be used in monitoring the growth of maize inbred lines.

(2) Among the parameter systems of the WOFOST model, the parameter set of maize inbred lines formed in this study mainly has eight parameters used to distinguish maize inbred lines from hybrids (Chapter 3 for details), and the differences and similarities in parameter values can be explained in terms of agronomic mechanisms and corroborated with the results of other studies.

(3) In this paper, two assimilation methods are proposed for estimating maize inbred lines yield: the assimilation method of inbred lines parameter set and the assimilation method of singe parameter. Compared with the two, the assimilation method of parameter set has more accurate and robust simulation results, and the algorithm simulates a larger dynamic range, which has been able to estimate maize inbred lines yield better.

(4) In the validation of yield estimation results, this study used plots as the basic unit for validation and matched the field yield measurement data with the assimilated yield estimation results at spatial scales to avoid errors caused by inconsistent data scales in the validation process, and from the validation results: R^2^ reached 0.56 and RMSE was 684.90 Kg/Ha.

The present study has the same limitations and shortcomings:

(1) First, in this study, we only obtained field observations of leaf area and phenological data of maize inbred lines in one year, and we will continue to obtain data for many years to continue the work of this study, to better calibrate the model, and to obtain more accurate growth parameters of maize inbred lines.

(2) This study did not compare the simulation effect of WOFOST model with other crop models, and it is still a question worth exploring whether other models can simulate the growth of maize inbred lines more easily and accurately.

(3) This study uses a single remote sensing data source, and the scale of the study is small. How to combine multiple sources of remote sensing data and apply the remote sensing yield estimation method of inbred lines developed in this study on a large scale, or even estimate the maize seed production capacity of the whole China, will be the focus of our subsequent work.

## Data availability statement

The original contributions presented in the study are included in the article/**Supplementary Material**, further inquiries can be directed to the corresponding author.

## Author contributions

The JL, YM, SC, XH, HW, SL, ZL, and XZ conducted the field experiment. JL conducted the image analysis. Other authors discussed, wrote the manuscript, and designed the experiments. All authors contributed to the article and approved the submitted version.
