# Supplementary material for: A method for estimating yield of maize inbred lines by assimilating WOFOST model with Sentinel-2 satellite data
Source: Front Plant Sci. 2023 Sep 7;14:1201179. doi: 10.3389/fpls.2023.1201179 (PMC10513754; doi:10.3389/fpls.2023.1201179)
Supplement: Supplementary file 1 [file Table_1.docx]

Supplementary Material

A method for estimating yield of maize inbred lines by assimilating WOFOST model with Sentinel-2 satellite data

Junyi Liu ^1^, Xianpeng Hou ^1^, Shuaiming Chen ^1^, Yanhua Mu ^1^, Hai Huang ^1^, Hengbin Wang ^1^, Zhe Liu ^1,2^*, Shaoming Li ^1,2^, Xiaodong Zhang ^1,2^, Yuanyuan Zhao ^1,2^, Jianxi Huang^1,2^

^1^College of Land Science and Technology, China Agricultural University, Beijing, China

^2^ Key Laboratory of Remote Sensing for Agri-Hazards, Ministry of Agriculture and Rural Affairs, Beijing, China

*** Correspondence:**Zhe Liu
liuz@cau.edu.cn

# Supplementary Figures and Tables

## Supplementary Tables

# Table.1 WOFOST crop parameters, their limits and descriptions

| Parameter name | Upper and lower bound | Definition | Unit |
| --- | --- | --- | --- |
| CVL | [0.6,0.76] | efficiency of conversion into leaves | Kg·Kg-1 |
| CVS | [0.63,0.76] | efficiency of conversion into stems | Kg·Kg-1 |
| CVR | [0.65,0.76] | efficiency of conversion into roots | Kg·Kg-1 |
| CVO | [0.45,0.85] | efficiency of conversion into storage org | Kg·Kg-1 |
| TBASE | [-10,10] | lower threshold temperature for ageing of leaves | ℃ |
| TEFFMX | [18,32] | maximum efficiency temperature for emergence | ℃ |
| TSUM1 | [150,1050] | temperature sum from emergence to anthesis | ℃·d |
| TSUM2 | [600,1550] | temperature sum from anthesis to maturity | ℃·d |
| TSUMEM | [0,170] | temperature sum from sowing to emergence | ℃·d |
| TDWI | [10,70] | initial total crop dry weight | Kg |
| SLATB000 | [0.0007,0.0042] | specific leaf area at 0.00 at the growth period | hm2·Kg-1 |
| SLATB078 | [0.0007,0.0042] | specific leaf area at 0.78 at the growth period | hm2·Kg-1 |
| SLATB200 | [0.0007,0.0042] | specific leaf area at 2.00 at the growth period | hm2·Kg-1 |
| SPAN | [17,50] | life span of leaves growing at 35 Celsius | d |
| RGRLAI | [0.007,0.5] | maximum relative increase in LAI | ha·ha-1·d-1 |
| LAIEM | [0.0007,0.30] | leaf area index at emergence | ha·ha-1 |
| RML | [0.027,0.030] | maintenance respiratory rate leaves | Kg·(Kg·d)-1 |
| RMO | [0.003,0.017] | maintenance respiratory rate storage organs | Kg·(Kg·d)-1 |
| Q10 | [1.5,2.0] | increase in respiratory rate per 10 ℃ temperature increase | / |
| AMAXTB000 | [1,70] | maximum leaf CO2 assimilation rate at 0.00 at the growth period | Kg·hm-2·h-1 |
| AMAXTB125 | [1,70] | maximum leaf CO2 assimilation rate at 1.25 at the growth period | Kg·hm-2·h-1 |
| AMAXTB150 | [1,70] | maximum leaf CO2 assimilation rate at 1.5 at the growth period | Kg·hm-2·h-1 |
| AMAXTB175 | [1,70] | maximum leaf CO2 assimilation rate at 1.75 at the growth period | Kg·hm-2·h-1 |
| AMAXTB200 | [1,70] | maximum leaf CO2 assimilation rate at 2.00 at the growth period | Kg·hm-2·h-1 |
| TMPFTB000 | [0.0,1.0] | Correction factor for the maximum CO2 assimilation rate at 0°C | / |
| TMPFTB900 | [0.0,1.0] | Correction factor for the maximum CO2 assimilation rate at 9°C | / |
| TMPFTB160 | [0.0,1.0] | Correction factor for the maximum CO2 assimilation rate at 16°C | / |
| TMPFTB180 | [0.0,1.0] | Correction factor for the maximum CO2 assimilation rate at 18°C | / |
| TMPFTB200 | [0.0,1.0] | Correction factor for the maximum CO2 assimilation rate at 20°C | / |
| TMPFTB300 | [0.0,1.0] | Correction factor for the maximum CO2 assimilation rate at 30°C | / |
| TMPFTB360 | [0.0,1.0] | Correction factor for the maximum CO2 assimilation rate at 36°C | / |
| TMPFTB420 | [0.0,1.0] | Correction factor for the maximum CO2 assimilation rate at 42°C | / |
| KDIFTB00 | [0.44,1] | extinction coefficient for diffuse visible light at 0 at the growth period | / |
| KDIFTB20 | [0.44,1] | extinction coefficient for diffuse visible light at 2 at the growth period | / |
| EFFTB00 | [0.4,0.5] | light-use efficiency single leaf at 0 °C | (Kg·hm-2·h-1) ·(J·m-2·s-1) |
| EFFTB40 | [0.4,0.5] | light-use efficiency single leaf at 40 °C | (Kg·hm-2·h-1) ·(J·m-2·s-1) |
